# An exploratory study of the need for curriculum review of Master of Public Health Degree at a Rural-based University in South Africa

**DOI:** 10.4102/phcfm.v8i2.993

**Published:** 2016-05-20

**Authors:** Takalani G. Tshitangano

**Affiliations:** 1 Department of Public Health, University of Venda, South Africa

## Abstract

**Background:**

Master of Public Health (MPH) training programmes were developed worldwide in response to the crisis in human resources for health.

**Aim:**

To determine whether the MPH programme at the selected rural-based university in South Africa enabled students to achieve the MPH core competencies relevant for Lower Middle Income Countries.

**Setting:**

The study was carried out at a rural-based University in South Africa. The target population was the 2011 first-year cohort of MPH students who by the beginning of 2014 had just completed their coursework.

**Methodology:**

A quantitative cross-sectional descriptive research design was adapted. Eighty-five students were randomly selected to participate in the study. A structured questionnaire comprising seven competency clusters was developed. The selected students completed a self-administered questionnaire. Only those students who signed consent forms participated in this study. The questionnaire was tested for construct validity and reliability using 10 students with similar characteristics to those sampled for the study. Microsoft Excel software was used to analyse the data descriptively in terms of frequency and percentages.

**Results:**

The students were confident of their competencies regarding public health science skills. Amongst these were analytical assessment, communication, community and inter-sectorial competencies as well as ethics. However, the students lacked confidence in context-sensitive issues, planning and management, research and development, and leadership competencies. Yet the latter is the backbone of public health practice.

**Conclusion and recommendation:**

There is a need for revamping public health curricula. In this respect, a follow-up study that builds a deeper understanding of the subject is needed.

## Introduction

Calhoun and Spencer et al. assert: ‘Numerous political, economic, social and technological factors currently influence globalization, which is also driving the current unprecedented interest and growth in world health and global workforce development prioritizing capacity building both within and across nations’.^1^ According to Calhoun and Spencer et al.:

in response to the crisis in human resources required to meet the world-wide demand for effective and efficient public health services, various Master of Public Health (MPH) training programs have been developed world wide. However, the long standing professional and geographic differences in MPH education and training programs, and roles and specialization requirements have compromised national health agenda globally.

Calhoun, Ramaih et al. further state that:

equally impacted has been consensus building regarding the development of MPH educational standards across universities. In response to the repeated calls for collaborations and inter-university education, a consensus has been reached regarding the core competencies of MPH students, and was validated across regions in recent decades.^2^

MPH competencies are defined as ‘as a unique set of applied knowledge, skills and other attributes grounded in theory and evidence for the broad practice of public health’.^2^ The Association of Schools of Public Health (ASPH) identified core competencies for the Master of Public Health (MPH) degree in graduate schools and programmes of public health.^3^ The ASPH represents the 40 accredited schools of public health in North America, with a combined faculty of more than 7500 and an annual enrolment of nearly 1000 students. According to the ASPH:

to equip graduates for analysis and consideration of solutions to public health problems at the community, institutional, and societal levels, the MPH curriculum in graduate schools and programs of public health has traditionally been organized around five core disciplines namely, biostatistics, epidemiology, environmental health science, health policy and management, and social and behavioral sciences.^3^

However, the ASPH identified seven cross-cutting disciplines, namely, communication and informatics, diversity and culture, professionalism, programme planning, public health biology and systems thinking. Both core discipline competencies as well as cross-cutting competencies were validated in high-income countries.^3^

According to Moser: ‘the following MPH core competencies were formulated and validated for low and middle-income countries (LMICs) in a study in which the University of Western Cape (South Africa) participated’.^4^

Despite the availability of the validated core competencies for LMICs, the selected rural-based university in South Africa continues to follow its MPH original programme comprising only discipline-specific competencies. The concern is that Moser who had more than 20 years of experience in hiring and supervising MPH graduates discovered a considerable variation in the depth and quality of MPH graduates’ skills and knowledge in competency areas relevant to public health practice that fall outside their major field.^5^ Thus, Moser argues that both discipline-specific as well as the interdisciplinary and/or cross-cutting core competencies are intended to produce MPH graduates who can reliably be expected to possess a fundamental set of skills, regardless of their major field because successful public health practice requires knowledge and skills outside any MPH graduates’ major field.^5^

However, it is not clear if the MPH programme at a selected university in South Africa covers the fundamental knowledge, attitudes and skills that every MPH student regardless of their major field should possess upon graduation. This study aimed at determining whether the MPH programme at the rural-based university in South Africa enables students to achieve the MPH core competencies relevant for LMICs.

## Purpose of the study

This study was undertaken to determine whether the MPH programme at a selected rural-based university in South Africa enabled students to achieve the MPH core competencies relevant for LMICs.

## Research methods and design

A quantitative cross-sectional descriptive design was adopted. Students who registered for the MPH degree for the first time from 2011 to 2013 and had just completed their coursework at the beginning of 2014 constituted the study population. Eighty-five students were randomly selected.

### Data collection and analysis

A structured data collection instrument comprising 7 competency clusters, namely Public health science skills (including analytical assessment competencies; Communication competencies; Context-sensitive issues; Community and inter-sectorial competencies; Planning and management competencies; and Leadership and systems thinking competencies) where participants were expected to rate themselves competent or incompetent, was developed. Various authors were consulted to ensure construct validity of the instrument.^6,7,8,9,10,11,12^ The instrument was pretested using nine honours students from the school of health sciences of the same institution to test the clarity of the instrument and to determine the duration of completing the questionnaire. Informed written consent was obtained from all the respondents, including those used in the pretest of the instrument.

The data collection instrument was e-mailed to each individual student from an anonymous e-mail address to minimise study biasness. Thus, data were collected using self-administered questionnaires. The response rate was 92% (*n* = 78). Data were analysed using Excel spreadsheet to determine the frequencies. Data were presented using bar charts and tables.

## Results

The results of this study are organised on the basis of the validated core competencies for LMICs.

Public health science skills including analytical assessment competencies

**FIGURE 1:**
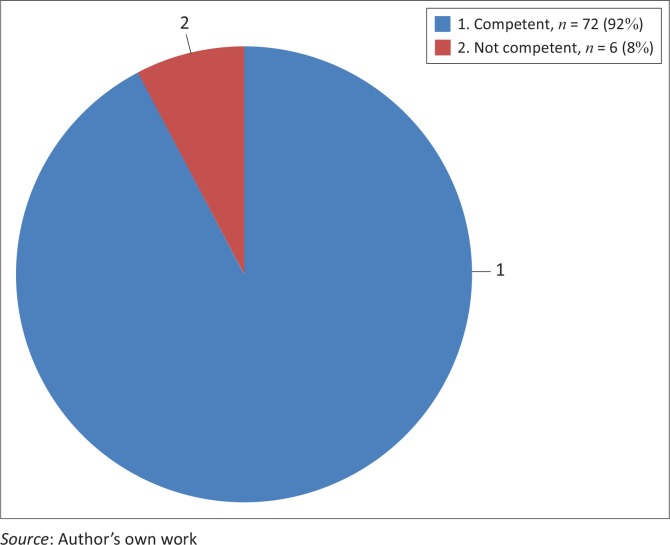
Students’ self-rating regarding public health science skills including analytical assessment competencies.

**FIGURE 2:**
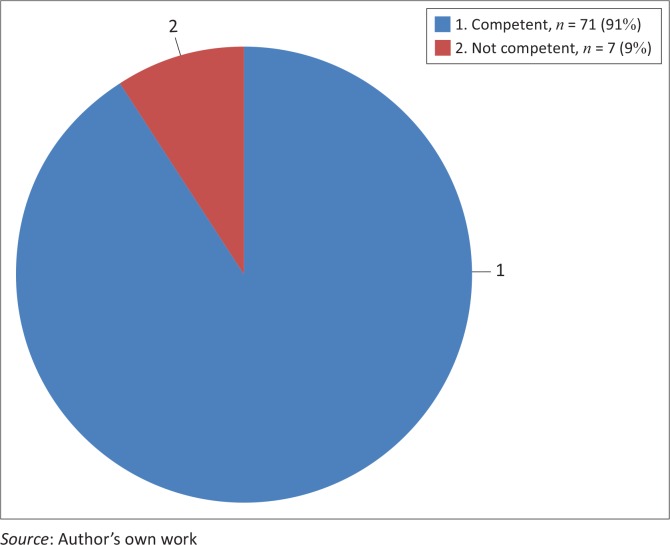
Students’ self-rating regarding their research and development competencies.

The majority of students (92%, *n* = 72) rated themselves competent on this core-competence as compared to only 6 students who rated themselves incompetent. In order to adequately assess this core-competence, the researcher added in the instrument an item named research and development, where about 91% (*n* = 71) students rated themselves incompetent on the core-competence. Figures 1 and 2 present the details.

### Communication competencies

Similarly, 92% of students rated themselves competent on this core-competence as compared to only 6 students who rated themselves incompetent (Tables 1 and 2).

**TABLE 1:** Validated Master of Public Health core competencies for low- and middle-income countries.

Cluster of competencies		Detailed competencies	
1.	Public health science skills including analytical assessment competencies.	1.	Applies the basic public health science (including but limited to biostatistics, epidemiology, environmental health services, health services administration and social and behavioural health sciences) to public health policies.
		2.	Appraise scope, function and role of public health in relation to local context, health system and other social sectors.
		3.	Assess population health status and identifies population health problems, risk factors, related social determinants and determine needs.
		4.	Commissions and critically interprets research findings and/or develops protocol and collects, analyses and synthesises reliable data using qualitative and quantitative methods.
2.	Policy development competencies	5.	Analyses and evaluates policy options and determines feasibility for public health policies/programmes in diverse community contexts, using appraisal of evidence.
		6.	Participates in developing context-sensitive policies and strategic plans and translates them into action.
3.	Communication competencies	7.	Understands and contributes to developing and using mechanisms to monitor and evaluate public health policies and regulations.
		8.	Contributes to advocacy of new and existing health policies to the public health and other sectors.
		9.	Communicates concisely in writing and orally, in person and through electronic means with linguistic cultural proficiency and appropriateness.
		10.	Facilitates and integrates input to public health policy and programmes from a range of individual and organisational stakeholders.
		11.	Uses a variety of culturally appropriate approaches to disseminate public health information with consideration to ethical and confidential issues.
4.	Context-sensitive issues	12.	Analyses the role of gender, cultural, social, economic, political and behavioural factors in the accessibility, availability, acceptability and delivery of public health service and programmes.
		13.	Incorporates ‘social determinants of health’ approach to public health needs.
5.	Community and inter-sectorial competencies	14.	Assesses and engages community actors and communities and their linkages and relationships that affect health in diverse social and cultural situations.
		15.	Collaborates in community-based participatory efforts.
		16.	Develops and maintains partnerships with key stakeholders, including from different sectors.
6.	Planning and management competencies	17.	Uses evidence and good practice to address public health policy, planning and management issues.
		18.	Plans, implements, monitors and evaluates public health interventions, programmes, and resources, services including inputs, process, outcome and impact.
		19.	Prepares and contributes to manage and evaluate public health information systems, human, financial and logistic resources.
7.	Leadership and systems thinking competencies	20.	Demonstrates leadership as a manager and in team efforts and is able to lead in public health emergencies.
		21.	Demonstrates professional judgements and ethical standards in data handling and addressing public health issues and diverse opinions.
		22.	Leads with applying the understanding of interconnectedness and dynamic interactions of the public health system.
		23.	Continues lifelong learning and professional development and stimulates team to do so.

*Source*: The council on linkages between academia and public health practice^5^

**TABLE 2:** Frequency and percentages of communication competencies of Master of Public Health students.

Communication	Frequency	Percentage
Competent	72	92
Not competent	6	8

*Source*: Author’s own work

### Context-sensitive issues

**FIGURE 3:**
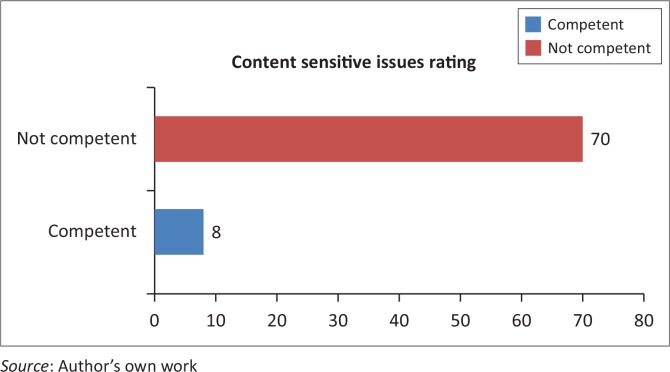
Students’ self-rating regarding their context-sensitive issues.

About 89% (*n* = 70) of students rated themselves incompetent on this co-competence as compared to few (*n* = 8) who rated themselves competent (Figure 3).

### Community and inter-sectorial competencies

The majority (98%) of students rated themselves competent in this core-competence, as compared to only 2% who rated themselves incompetent (Table 3).

**TABLE 3:** Frequency and percentages of community and inter-sectorial competencies of Master of Public Health students.

Communication	Frequency	Percentage
Competent	77	98
Not competent	1	2

*Source*: Author’s own work

### Planning and management competencies

**FIGURE 4:**
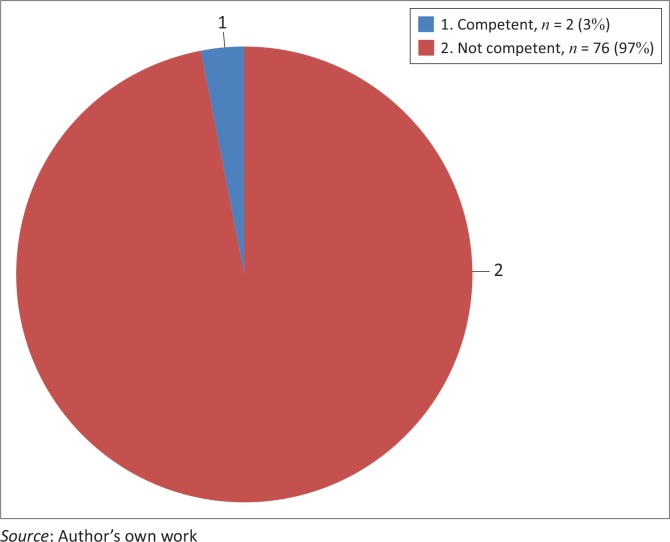
Students’ self-rating regarding their planning and management competencies.

About 97% (*n* = 76) of students rated themselves incompetent in this core-competence, as compared to 3% who rated themselves competent (Figure 4).

### Leadership and systems thinking competencies

**FIGURE 5:**
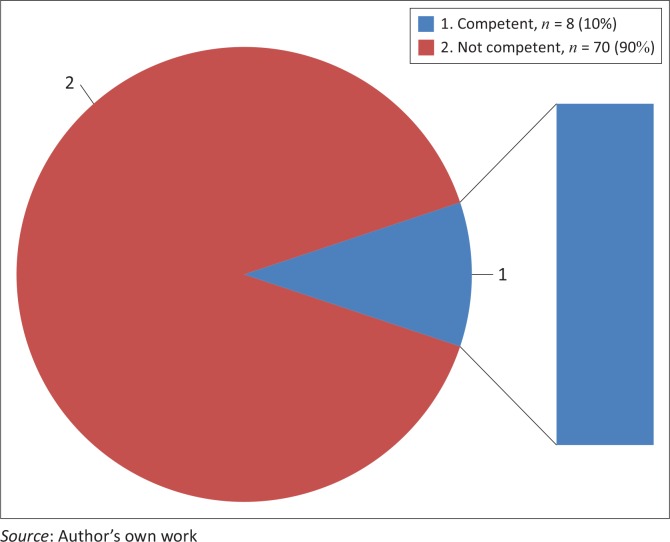
Students’ self-rating regarding their leadership and systems thinking competencies.

About 89% (*n* = 70) of students rated themselves incompetent on this co-competence, as compared to few (*n* = 8) who rated themselves competent. The researcher further added an item on the instrument to measure students’ competence on management of self, people and resources ethically, where the majority (*n* = 74) of students rated themselves competent. Figure 5 presents the details.

## Discussion

The results of this study show that students at a selected university in South Africa are confident of their competency regarding public health science skills, including analytical assessment competencies, communication competencies, community and inter-sectorial competencies, and ethics. However, the study has revealed that students still lack confidence on context-sensitive issues, planning and management competencies, research and development, and leadership competencies, which are the backbone of the public health practice. This means that the programme at the selected university is putting much effort on imparting skills and knowledge on the graduate’s major field neglecting fundamental skills that a successful public health practitioner requires.

The results of this study are similar to those of the situational assessment of the MPH programmes at the University of Toronto, which identified curriculum gaps in seven competencies, namely:^13^

- Develop strategies to motivate others for collaborative problem solving, decision making and evaluation.
- Use current technology to communicate effectively.
- Use skills such as team building, negotiation, conflict management and group facilitation to build partnership.
- Evaluate an action, policy or program.
- Demonstrate an ability to set and follow priorities, and to maximise outcomes based on available resources.
- Apply principles of program planning, development, budgeting, management and evaluation in organisational and/or community initiatives.
- Demonstrate knowledge of Canada’s public health systems (e.g. federal, provincial and local).

### Limitations of the study

The study was conducted amongst MPH students registered at only one South African University. Thus, generalisability of the findings is limited to this institution only.

## Conclusion and recommendations

There are serious gaps in the MPH degree curriculum at the selected rural-based university in South Africa. The gaps in the competencies have implications for curriculum, in the form of curriculum review in order to ensure that the MPH curriculum of the selected university in South Africa and the overall educational experience prepares our graduates to adapt and excel in their discipline-specific fields as well as the cross-cutting fields in order to tackle the public health issues of today and the future effectively.

The selected university should adopt the vision and mission statements together with the goals and objectives of the MPH suggested by public health associations for LMICs worldwide. The 23 core competencies should be adopted alongside the seven core competency clusters. In-depth consultation with lecturers and students, including alumni, for specific feedback to inform the curriculum should be considered. Issues surrounding practical should also be considered. Discipline-specific competencies for all fields in the MPH programme as well as strategies to address gaps in the current curriculum should be identified.
